# Speciation with gene flow: Evidence from a complex of alpine butterflies (*Coenonympha*, Satyridae)

**DOI:** 10.1002/ece3.5220

**Published:** 2019-05-03

**Authors:** Thibaut Capblancq, Jesús Mavárez, Delphine Rioux, Laurence Després

**Affiliations:** ^1^ Laboratoire d’Écologie Alpine UMR5553 CNRS‐Université Grenoble Alpes Grenoble France

**Keywords:** *Coenonympha*, evolutionary history, gene flow, HINDEX, speciation

## Abstract

Until complete reproductive isolation is achieved, the extent of differentiation between two diverging lineages is the result of a dynamic equilibrium between genetic isolation and mixing. This is especially true for hybrid taxa, for which the degree of isolation in regard to their parental species is decisive in their capacity to rise as a new and stable entity. In this work, we explored the past and current patterns of hybridization and divergence within a complex of closely related butterflies in the genus *Coenonympha* in which two alpine species, *C. darwiniana* and *C. macromma*, have been shown to result from hybridization between the also alpine *C. gardetta* and the lowland *C. arcania*. By testing alternative scenarios of divergence among species, we show that gene flow has been uninterrupted throughout the speciation process, although leading to different degrees of current genetic isolation between species in contact zones depending on the pair considered. Nonetheless, at broader geographic scale, analyses reveal a clear genetic differentiation between hybrid lineages and their parental species, pointing out to an advanced stage of the hybrid speciation process. Finally, the positive correlation observed between ecological divergence and genetic isolation among these butterflies suggests a potential role for ecological drivers during their speciation processes.

## 1 INTRODUCTION

Hybridization between diverging taxa is possible throughout the speciation process until complete reproductive isolation is achieved (Abbott et al., 2013; Butlin, Galindo, & Grahame, 2008; Descimon & Mallet, 2009; Nosil, 2012). In some cases, the genetic exchanges favor the development of a hybrid population, which can take advantage of new combinations of traits resulting from the rearrangement of parental phenotypes and rise itself as a distinct hybrid species (Mallet, 2007; Mavárez & Linares, 2008; Rieseberg, 1997; Seehausen, 2004). The determinant step for hybrid speciation is the achievement of isolation between the new‐born hybrid lineage and the two parental genetic pools (Abbott, Hegarty, Hiscock, & Brennan, 2010; Mallet, 2007; Nieto Feliner et al., 2017; Petit & Excoffier, 2009; Schumer, Rosenthal, & Andolfatto, 2014). However, speciation is not a punctual phenomenon but a continuous process over time, at the beginning of which the hybrid lineage is not expected to be fully reproductively isolated from the parental species and the two types of populations, hybrid and parental, likely remain interconnected to some degree. This situation has sometimes been called a “hybrid swarm” (Jiggins & Mallet, 2000; Seehausen, 2004) to illustrate the tenuous equilibrium between isolation and mixing that prevails in. The hybrid swarm phase would vary from a few to hundreds of generations depending on the rate at which the hybrid lineage builds up reproductive isolation (RI), a process that can be greatly enhanced by the adaptation of the hybrid lineage to environmental conditions distinct from those of the parental lineages (Abbott et al., 2010; Buerkle, Morris, Asmussen, & Rieseberg, 2000; Mallet, 2007; Meier et al., 2017; Nosil, Egan, & Funk, 2008; Rieseberg et al., 2003). Later on, when isolating reproductive barriers have evolved, this hybrid swarm phase ends and a hybrid speciation phase becomes possible (Schumer et al., 2014; Seehausen, 2004).

When viewing speciation as a continuous process its progress can be evaluated by the strength of the isolation between the diverging lineages (Butlin et al., 2008; Hendry, Bolnick, Berner, & Peichel, 2009; Mallet, 2008; Petit & Excoffier, 2009). The more the lineages are genetically connected, the less they have reached an advanced stage in the speciation continuum and the less they are susceptible to remain distinct in the future as independent genetic entities (Hendry et al., 2009). The strength of gene flow between taxa, both during their past history of divergence and currently in contact zones, can therefore be used as a proxy for reproductive isolation and serve as a way to settle the state of the speciation process.

**Figure 1:**
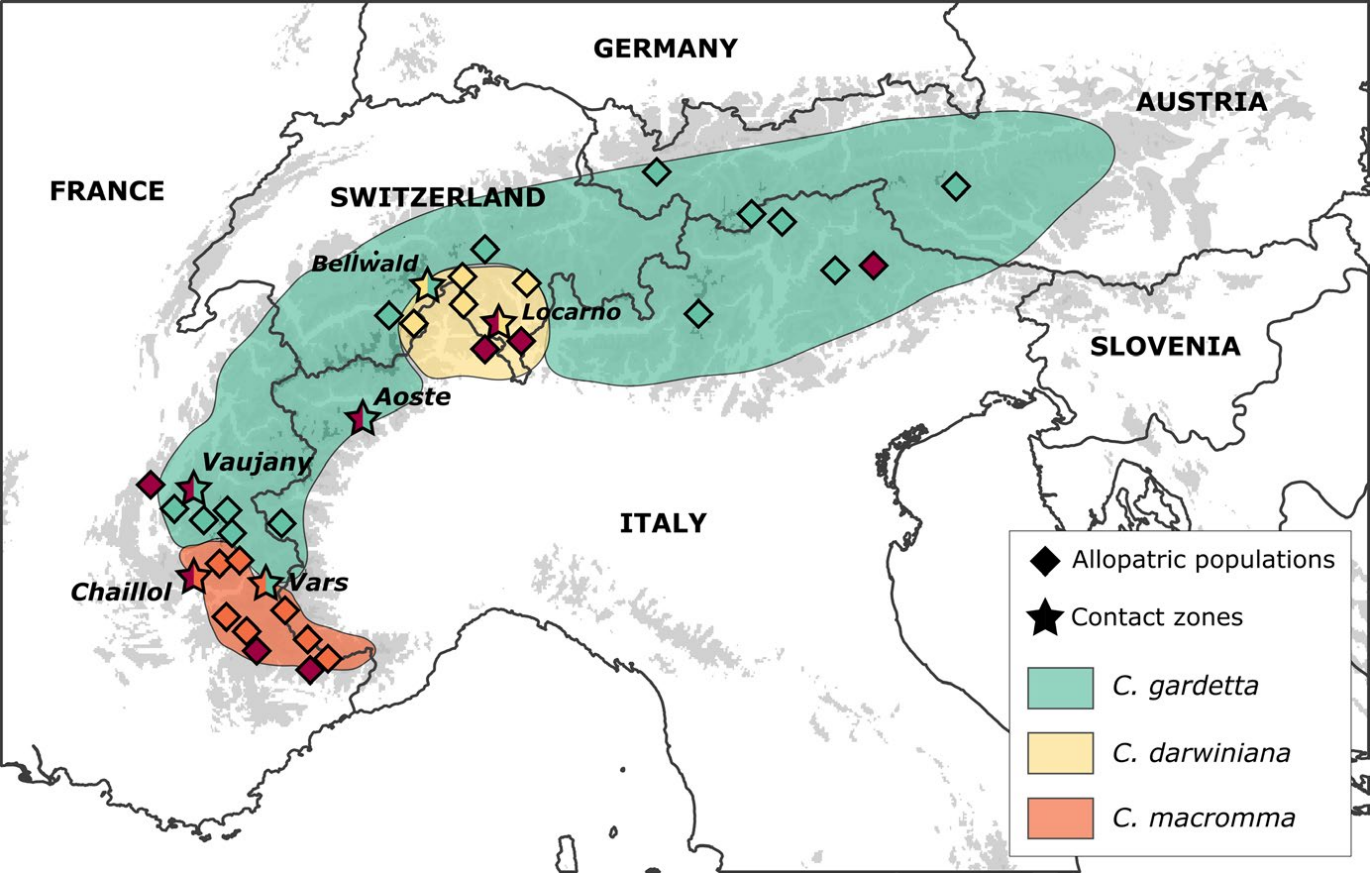
Distribution range of the three alpine *Coenonympha* species: *C. gardetta*,* C. macromma,* and *C. darwiniana*. The range of the fourth species, *C. arcania* (everywhere in Europe at low elevation, including the valleys of the Alps) is not shown. Stars: location of contact zones *C. arcania/C. macromma* in Chaillol, *C. arcania/C. gardetta* in Vaujany and Aoste, *C. arcania/C. darwiniana* in Locarno, *C. gardetta/C. darwiniana* in Bellwald, and *C. gardetta/C. macromma* in Vars. Diamonds: locations of allopatric populations: *C. arcania* (dark red), *C. gardetta* (blue‐green), *C. macromma* (orange), and *C. darwiniana* (pale yellow)

In a previous work, we studied a complex of butterfly species in the genus *Coenonympha* (Nymphalidae, Satyrinae), and found, using molecular markers (ddRADseq) and an Approximate Bayesian Computation (ABC) framework, that *C. darwiniana* (Staudinger 1871) and *C. macromma* (Turati & Verity 1910) are the product of hybridization between the Pearly Heath *C. arcania* Linné 1761 and the Alpine Heath *C. gardetta* Prunner 1798 (Capblancq, Després, Rioux, & Mavárez, 2015). The two parental species are good examples of adaptive diversification along an altitudinal gradient, with *C. arcania* being widely distributed in Europe from sea level to elevations around 1,500 m, while *C. gardetta* is typically encountered above 1,500 m in the Alps, the French Massif Central and the Balkans. The two hybrid lineages are also found at relatively high elevations (1,300–2,500 m) and they prosper in similar alpine climatic conditions as *C. gardetta,* which they replace in two distinct geographic areas (see Figure 1). We also suggested in this previous study that the two hybrid taxa originated from a unique ancestral hybrid population ~10,000–20,000 years ago (Capblancq et al., 2015), after which they diverged from each other rapidly, probably with the establishment in the two allopatric geographic areas they currently occupy (see Figure 1). The two hybrid species can sometimes be found flying with parental species in narrow zones at either intermediate elevations (with *C. arcania*) or where distribution ranges abut (with *C. gardetta*).

The objectives of the present study were to explore in further depth the scenarios for the evolution of the species in this complex, by taking into consideration additional processes such as interspecific gene flow during divergence, genomic heterogeneity in introgression rates and changes in effective population size through time. We also aimed to evaluate the advancement of the different speciation processes within the complex and, especially, in the two hybrid lineages: *C. macromma* and *C. darwiniana*. We took advantage of our previous study sampling: 130 individuals in 30 allopatric populations across the range of the four species; and, in order to increase the possibility to detect genetic exchanges between species, we sampled six new locations in which two species were found flying together, hereafter called contact zones. We used a dataset of SNP genotypes obtained from a double‐digested RAD‐seq library to infer the histories of divergence among species using their joint allele frequency spectrum (JAFS). We modeled and tested several past scenarios of divergence and gene flow to evaluate the most likely direction, degree and timing of hybridization during the evolution of the complex. We then confronted these past scenarios against the current reproductive isolation among the four taxa inferred from patterns of hybridization in contact zones. More specifically, we looked for evidence of both past genetic mixing among species in the complex at large geographic scale and current hybridization between species pairs at fine geographic scale in contact zones. Finally, we evaluated the relation between gene flow and patterns of morphological, genetic, and ecologic differentiation among the species in the complex to understand the possible influence of these factors in the speciation of the hybrid taxa.

## 2 MATERIALS AND METHODS

### 2.1 Butterfly sampling

A total of 301 individuals of the four butterfly species were sampled in 36 localities along the Alps, with a particular sampling effort in six locations where two species can be found together (hereafter “contact zones”) and nearby locations where only one species is observed (hereafter “allopatric populations”; Table 1 and Figure 1). Among these individuals, 130 were collected in allopatric populations during a previous study on the complex (Capblancq et al., 2015) and 171 are new samples from the six contact zones. Samples were kept in glassine envelopes in the field and in ethanol 96% at −20°C in the laboratory until DNA extraction.

**Table 1:** Sampling locations and their characteristics

Locality	Number of individuals	Species	Country	Latitude	Longitude
Allopatric populations					
Orcières	3	*C. arcania*	France	44.71	6.33
Colmars	3	*C. arcania*	France	44.23	6.63
Monte Baro	3	*C. arcania*	Switzerland	46.09	9.00
Cortina d'Ampezzo	6	*C. arcania*	Italy	46.56	12.12
Mont Jalla	4	*C. arcania*	France	45.20	5.72
Viggiona	2	*C. arcania*	Italy	46.04	8.68
Saint Dalmas	3	*C. arcania*	France	44.07	7.19
Ailefroide	5	*C. gardetta*	France	44.90	6.44
Albergpass	5	*C. gardetta*	Austria	47.14	10.20
Ornon	5	*C. gardetta*	France	45.05	5.93
Passo Gardena	5	*C. gardetta*	Italy	46.53	11.78
Passo Giovo	5	*C. gardetta*	Italy	46.83	11.31
Heilingblunt	5	*C. gardetta*	Austria	47.05	12.85
Lautaret	4	*C. gardetta*	France	45.04	6.40
Moosalps	5	*C. gardetta*	Switzerland	46.25	7.83
La Selle	5	*C. gardetta*	France	44.98	6.19
Oberalpspass	5	*C. gardetta*	Switzerland	46.66	8.68
Solden	5	*C. gardetta*	Switzerland	46.88	11.04
Sestrière	5	*C. gardetta*	Italy	44.96	6.88
Passo Tonale	5	*C. gardetta*	Italy	46.26	10.57
Boreon	4	*C. macromma*	France	44.12	7.29
Dormillouse	2	*C. macromma*	France	44.73	6.45
Foux d'Allos	4	*C. macromma*	France	44.29	6.57
Col de Larche	4	*C. macromma*	France	44.42	6.91
Col de la Lombarde	4	*C. macromma*	Italy	44.24	7.11
Seynes	4	*C. macromma*	France	44.38	6.39
All'Acqua	4	*C. darwiniana*	Switzerland	46.49	8.48
Bosco‐Gurin	4	*C. darwiniana*	Switzerland	46.32	8.49
Fontane	4	*C. darwiniana*	Switzerland	46.45	9.05
Simplon Dorf	4	*C. darwiniana*	Switzerland	46.20	8.05
Contact zones					
Aosta	21	*C. arcania ‐ C. gardetta*	Italy	45.63	7.61
Vaujany	30	*C. arcania ‐ C. gardetta*	France	45.17	6.09
Locarno	31	*C. arcania ‐ C. darwiniana*	Switzerland	46.20	8.79
Chaillol	33	*C. arcania ‐ C. macromma*	France	44.68	6.16
Bellwald	26	*C. gardetta ‐ C. darwiniana*	Switzerland	46.43	8.16
Vars	30	*C. gardetta ‐ C. macromma*	France	44.53	6.70

### 2.2 Genetic data acquisition

DNA was extracted from the complete thorax of each individual using the DNeasy Blood and Tissue Kit (QIAgen, Germany). A dataset of single‐nucleotide polymorphisms (SNPs) was produced using double‐digested restriction‐site associated DNA (ddRAD) sequencing using a modified version of the protocol in Peterson, Weber, Kay, Fisher, and Hoekstra (2012) described in Capblancq et al. (2015). Six different *Sbf*I/*Msp*I ddRAD libraries were sequenced, each one in 1/10 of lane of a HiSeq 2500 Illumina sequencer (Fasteris S.A., Switzerland). The obtained DNA reads (~60 million of 2 × 125 paired‐end reads) were used to call SNP genotypes, using de novo assembling, with the *STACKS* pipeline (Catchen, Hohenlohe, Bassham, Amores, & Cresko, 2013). We used a Phred score of 10 for reads filtering (*process_radtags* function), a minimum coverage of five reads to create a stack (−m 5 in *ustacks* function) and a maximum of 6 different nucleotides to merge two different stacks (−M 6). Highly repetitive and over‐merged stacks were dropped using both the “Removal algorithm” and the “Deleveraging algorithm.” Furthermore, a maximum of 10 mismatches was allowed for considering two individual tags as the same locus and to merge them in the catalog (−n 10 in the *cstacks* function). Finally, only one SNP per polymorphic stack, on RAD‐tags present in at least three of the four species, in more than 40% of the sampling and with a frequency higher than 1% of the total sampling was used for further analyses. The libraries produced a mean of 3200 RAD‐tags with a mean coverage of 35 reads/tag for the 301 individuals analyzed, resulting in a genotype matrix including 1,047 SNPs.

### 2.3 History of divergence and gene flow among species

**Figure 2:**
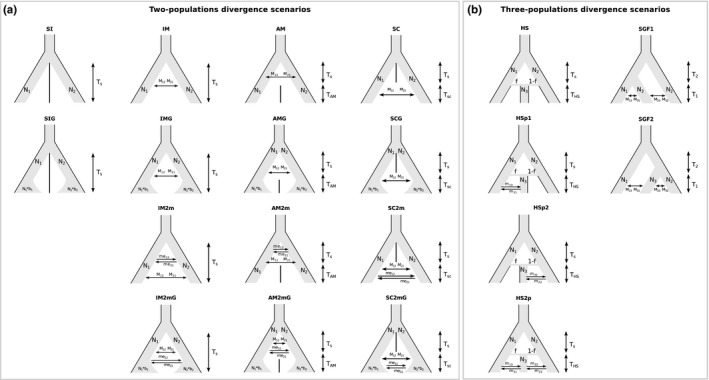
Evolutionary scenarios tested through the ∂a∂I procedure with two‐population divergence (a) and three‐populations divergence (b) cases. In (a), the first row represents the basic scenarios: strict isolation (SI), isolation with migration (IM), ancient migration (AM), secondary contact (SC). The second row represents the same scenarios plus population growth (SIG, IMG, AMG, SCG), the third row shows scenarios allowing heterogeneous migration rate along the genome (IM2 m, AM2 m, SC2 m) and the fourth row details the more complex models with both population size change and heterogeneous gene flow (IM2 mG, AM2 mG, SC2mG). In (B) are described the four tested models of hybrid speciation (HS, HSp1, HSp2, and HSp2) and the two tested models of secondary gene flow (SGF1 and SGF2)

To determine the influence of interspecific hybridization on the pattern and timing of genomic differentiation of species in the complex, the demographic divergence of the lineages was inferred using their joint allele frequency spectrum (JAFS) and the likelihood approach implemented in ∂a∂I (Gutenkunst, Hernandez, Williamson, & Bustamante, 2009). We used only the allopatric populations and the 1,047 SNPs obtained with the ddRAD sequencing to build a JAFS between each pair of species using R scripts from https://github.com/laninsky/creating_dadi_SNP_input_from_structure. The six different JAFS were then projected down to 15 individuals per species to avoid missing genotypes and optimize the resolution of the analyses. We first tested for different evolutionary scenarios of speciation of *C. macromma* and *C. darwiniana* using two types of models that produce a hybrid lineage: hybrid speciation, *that is,* a punctual mixing of two parental populations gives birth to a third taxon, and secondary gene flow, that is, the future hybrid taxon initially diverged from one of the parental species before hybridizing with the other (see Schumer et al., 2014). To do so, we used four modified versions of the hybrid speciation model (HS) and two modified versions of the secondary gene flow model (SGF) designed in Eaton, Hipp, González‐Rodríguez, & Cavender‐Bares, 2015 (see Figure 2): no further gene flow between hybrid and parental lineages after the original hybrid speciation event (HS), gene flow with the two parental species after the original hybrid speciation event (HS2p), gene flow with one or the other parental species after the original hybrid speciation event (HSp1 and HSp2), divergence from *C. arcania* followed by gene flow with both parental species (SGF1), and divergence from *C. gardetta* followed by gene flow with both parental species (SGF2). We tested separately the evolutionary scenario of *C. macromma* and *C. darwiniana* even if previous work on these species points out their common origin (Capblancq et al., 2015). We did so because ∂a∂I does not allow the simultaneous analysis of more than three different populations but also because this strategy reduces the complexity of the models and allows the focusing of the analysis on the independent history of interactions between each hybrid taxon and their two parental species.

We also tested for alternative models of “paired‐species” divergence between *C. arcania* and *C. gardetta* and between *C. macromma* and *C. darwiniana* (see Figure 2), using four basic models of divergence designed in Rougeux, Bernatchez, & Gagnaire, 2017 and Tine et al., 2014: strict isolation (SI) (i.e., no gene flow after divergence), isolation with migration (IM) (i.e., continuous gene flow after divergence), ancient migration (AM) (i.e., gene flow during divergence, not afterward), and secondary contact (SC) (i.e., strict isolation during divergence, gene flow afterward); and ten additional models derived from these basic four: four models that account for temporal variation in the effective population size of diverging lineages (SIG, IMG, AMG, SCG), three models that allow for genomic variations in the effective rate of gene flow along the genome, thereby simulating differential levels of genomic introgression (IM2m, AM2m, SC2m), and three models that take both temporal variation in effective population size and heterogeneous gene flow into account (IM2mG, AM2mG, SC2mG).

All these evolutionary models were fitted independently for each pair or triplet of species using “BFGS” optimization (Gutenkunst et al., 2009). We ran 10 independent optimizations for each model, keeping in each case only the run with the highest likelihood value. Then, we retained the model with the lowest Akaike information criterium (AICmin) and all the models with AIC − AIC_min_ < 10 (Burnham & Anderson, 2004; Rougeux et al., 2017). Finally, as described in Rougeux et al., 2017, we used the difference between the worst and the best models: ∆_max_ = AIC_max_ − AIC_min_ to calculate a model score = (∆_max_ − ∆AIC_i_)/∆_max_ for each of the alternative models and each pair or triplet of species. To evaluate the relative probabilities of the different models, we also computed Akaike weights (wAIC) following the equation described in Rougeux et al., 2017. The demographic parameters were estimated from the retained scenarios (AIC − AIC_min_ < 10) for each pair or triplet of species, and their values were used to compare the timing and strength of gene flow among species.

### 2.4 Current genetic structure and differentiation among the populations

We analyzed the population structure and genomic admixture among allopatric populations of the species in the complex using the Bayesian clustering method implemented in *STRUCTURE* 2.3 (Pritchard, Stephens, & Donnelly, 2000). Assignment of individuals to genetic clusters was accomplished using the 1,047 SNPs dataset obtained with the ddRAD sequencing described above. No prior population information was provided, and three independent runs of 500,000 generations were done for a number of clusters ranging from *K* = 2 to 9 and with a burnin period of 50,000 generations. The run with the highest likelihood was kept for each *K* value, and its confidence was assessed by looking at the inter‐run variability of the likelihood. All the individuals from allopatric populations were analyzed in the same *STRUCTURE* project in order to correctly infer their probabilities of ancestry. Finally, the genetic differentiation of the taxa was assessed by calculating the *F*_st_ between each pair of species according to Nei's method (Nei, 1987) with the R‐package *adegenet* (Jombart, 2008).

### 2.5 Individual index of hybridization and interspecific heterozygosity estimation in contact zones

An estimation of the genetic admixture was calculated for every individual within each of the six contact zones using the maximum‐likelihood procedure implemented in the R‐package *introgress* (Gompert & Buerkle, 2010). This software provides a genetic hybrid index (HINDEX) representing an estimate of the proportion of alleles that were inherited from one of the two parental species (Buerkle, 2005). Reference values for allele frequencies were established for each parental species using individuals from allopatric populations. Each contact zone was analyzed considering all the individuals as potential hybrids between the two parental allele frequency references depending on the taxa in contact at the location. The HINDEX ranges from 0 to 1, with extreme values corresponding to pure individuals of each reference parental species. This method allows for the use of codominant markers and, quite appropriately for closely related species, for markers that are not necessarily fixed between taxa (Buerkle, 2005).

Interspecific heterozygosity was also estimated for each individual within each contact zone. To do so, we used the function *calc.intersp.het* of the *introgress* R‐package, which estimates the proportion of the genome coming from each parental species (Buerkle, 2005). It gives an estimation of the direct bi‐ancestrality of each sampled genotype and can thus be used to detect recent hybrids (e.g., F1, F2, backcrosses), for which heterozygosity is expected to be higher than parental individuals. In contrast, in populations where admixture took place a long time ago and with no contemporary interspecific gene flow, hybrid individuals are not expected to show high heterozygosity levels but rather a mosaic of homozygous loci for alleles from either reference population.

### 2.6 Indexes of morphological and ecological differentiation among species

An index of morphological distances among populations was calculated from a geometric morphometric analysis of wing shape and patterns. 510 specimens were compared, including the 130 individuals used for genetic analyses of the allopatric populations in this study and additional samples from the same locations used in Capblancq et al. (2015). Morphometric distances among groups were estimated by calculating Mahalanobis’ distances on the scores of the discriminant analysis (linear discriminant analysis, LDA) performed on 22 forewing and 18 hindwing landmarks describing wing venation and pattern shape (see Appendix S1). All the analyses were accomplished using functions from the unpublished R‐package *RMORPH* (available under request to M. Baylac, MNHN, Paris, France).

In addition, an index of ecological differentiation was calculated from species occurrences and spatial climatic data. The climatic niche comparison was accomplished by extracting, for each occurrence data used in Capblancq et al. (2015) (6,900 points for *C. arcania*, 4,000 points for *C. gardetta*, 640 for *C. macromma,* and 400 points for *C. darwiniana*), the values of five noncorrelated “Bioclimatic” variables of precipitation and temperature: precipitation seasonality, annual precipitation, annual mean temperature, mean diurnal temperature range, and annual temperature range (http://worldclim.org). We assumed that the current climatic conditions experienced by a species provide good proxies of its abiotic ecological capacities and requirements (i.e., ecological envelope). We used the R‐package *ecospat* (Di Cola et al., 2017) to estimate niche similarity between each pair of taxa using the Schoener's overlap metric D (Broennimann et al., 2012; Schoener, 1970).

## 3 RESULTS

### 3.1 Scenarios of species divergences

#### 3.1.1 Divergence between parental species

**Figure 3:**
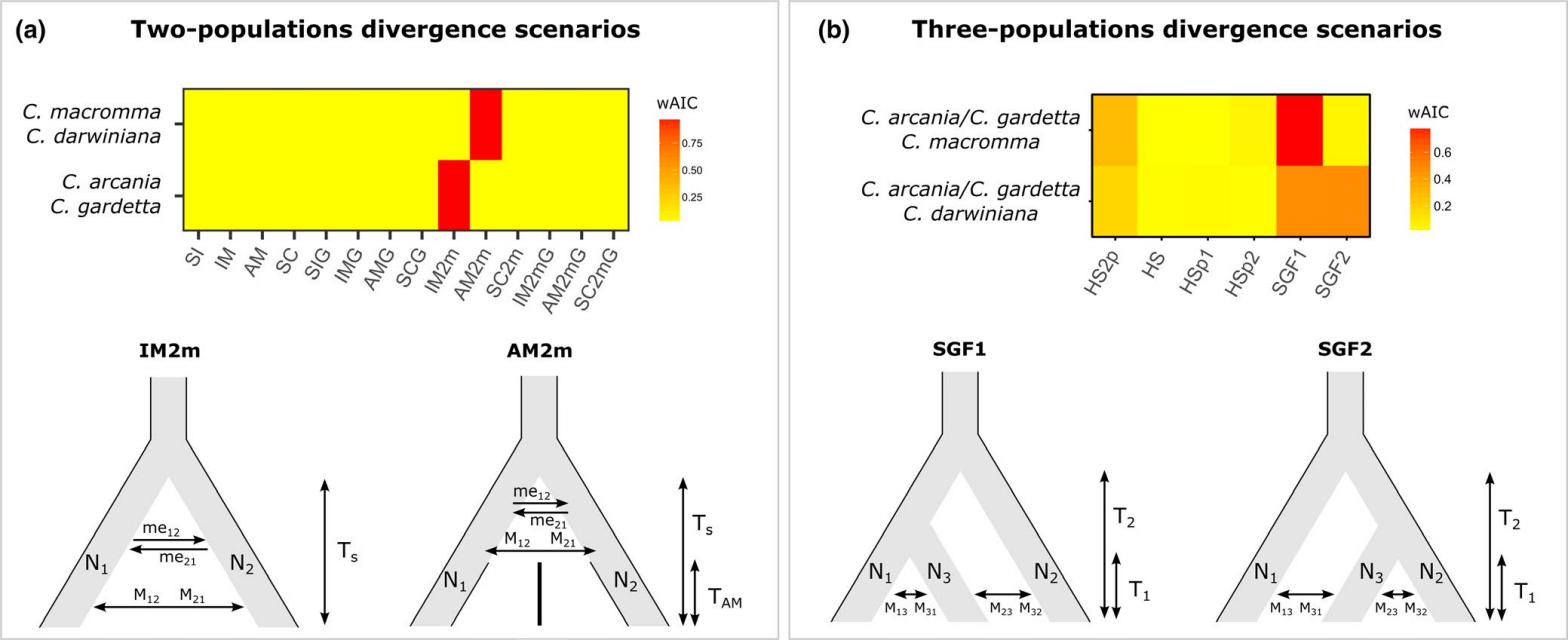
Demographic inferences using various evolutionary scenarios among *Coenonympha* species, including models of two‐population divergence (a) and three‐population divergence (b). Top: the wAIC resulting from ∂a∂I analyses of alternative scenarios. Bottom: the retained scenario for each species pairs or triplet

The best and only retained evolutionary model for the divergence between the parental species, *C. arcania* and *C. gardetta,* involves divergence with heterogeneous gene flow along the genome since the split between the lineages (IM2m) (Figure 3a and Table 2). At the opposite, the standard demographic models assuming homogeneous gene flow along the genome (i.e., Isolation with Migration, IM; and Ancient Migration, AM) do not satisfactorily reproduce the species joint allele frequency spectrum, even when population growth was allowed (IMG, AMG). In the same way, models involving a strict isolation all along the divergence process (SI, SIG) or a secondary contact between species (SC, SCG, SC2m, SC2mG) showed low probability scores and were not retained either (Figure 3, Table 2). The parameter estimates from the best model indicate a population size larger for *C. arcania* than for *C. gardetta,* which is expected given its wider geographical distribution, and an introgression rate far more important from *C. arcania* to *C. gardetta* (Table 2).

**Table 2:** Parameters estimates obtained for the evolutionary scenarios retained by the ∂a∂I analysis (∆AIC < 10)

Species	MODEL	MLE	AIC	deltaAIC	wAIC	theta	N1	N2	M12	M21	me12	me21	Ts	Tam	P	O
*C.arcania ‐ C.gardetta*	IM2m	−31.31	80.63	–	1	9.88	2.7	0.98	15.91	2.42	0.25	0.66	2.62	‐	0.31	0.68
*C.macromma ‐ C.darwiniana*	AM2m	−54.81	129.63	–	1	31.85	1.8	2.43	3.57	9.71	0.26	0.33	1.78	0.08	0.37	0.9

Species	MODEL	MLE	AIC	deltaAIC	wAIC	theta	N1	N2	N3	TH‐T2	TS‐T1	m13	m31	m23	m32	f
*C.arcania/C.gardettaC. macromma*	SGF1	−234.1	486.2	–	0.66	34.3	0.49	0.49	1.124	0.27	0.42	2.16	0.17	1.14	0.45	–
	HS2p	−234.1	488.2	2	0.24	34.2	0.494	0.489	1.126	0.26	0.44	2.13	0.19	1.15	0.43	1
	HSp2	−237.8	491.7	3.5	0.14	35.7	0.784	0.5	0.974	0.27	0.2	–	–	1.14	0.45	1
	SGF2	−236.9	491.8	5.6	0.04	32.5	0.53	0.58	0.913	0.001	0.59	1.75	0.55	0.78	0.38	–
	HS	−241.9	495.9	7.7	0.02	37.8	0.642	0.607	0.931	0.14	0.17	–	–	–	–	0.8
*C.arcania/C.gardettaC. darwiniana*	SGF2	−240.1	498.1	–	0.4	35.4	0.453	0.38	0.95	0.001	0.4	1.11	0.41	1.60	0.35	–
	SGF1	−240.1	498.1	0.01	0.4	35.3	0.454	0.38	0.956	0.001	0.40	1.10	4.09	1.60	0.35	–
	HS2p	−240.1	500.1	2	0.76	35.5	0.453	0.379	0.955	0	0.4	1.11	0.41	1.62	0.35	0.6
	HSp1	−244	504	3.9	0.11	39.3	0.377	0.504	0.914	0.07	0.2	1.16	0.42	–	–	0
	HS	−246.4	504.8	6.7	0.07	40.6	0.454	0.423	0.815	0.06	0.16	–	–	–	–	0.4
	HSp2	−244.7	505.4	7.3	0.05	40.1	0.497	0.315	0.882	0.05	0.19	–	–	1.62	0.04	0.7

#### 3.1.2 Divergence between hybrid species

The retained model of divergence between the two hybrid species, *C. macromma* and *C. darwiniana*, involves a primary period of gene flow just after the split, followed by a complete isolation (AM2m). The retained model suggests therefore that *C. macromma* and *C. darwiniana* do not exchange genetic material anymore, which is expected because these two species have disjoint distributions. As for the case of the divergence between parental species, adding the possibility of heterogeneous gene flow along the genome during the divergence process greatly increases the fit of the model for the hybrid species (Figure 3a).

#### 3.1.3 Scenarios of hybrid speciation

Concerning the scenarios of speciation for *C. macromma* and *C. darwiniana*, the different tested models return very close AIC values (Table 2), highlighting the difficulty to unmistakably select one of the analyzed models. Nonetheless, the model of secondary gene flow with *C. gardetta* following an initial divergence from *C. arcania* (SGF1) shows the lowest AIC for both *C. macromma* and *C. darwiniana* (Figure 3b). Furthermore, other models implying recurrent gene flow with parental species (e.g., SGF1, SGF2, and HS2P) also return low AIC values for the two hybrid species (Figure 3b), suggesting that allowing migration along the speciation process increases the fit of the models. Within each triplet, the parameters estimates are similar across retained models for population sizes (N1, N2, and N3), parental species split time (T_s_ or T_2_), migration rates (m13, m31, m23, m32) and admixture rate for HS scenarios (f). Only the timing of hybrid species birth (T_H_ or T_1_) can strongly vary from 0.27 (corresponding to half of the parental divergence) to 0.001 (Table 2). Interestingly, estimates of effective population size in *C. macromma* and *C. darwiniana* (N3) are approximately two times larger than the estimates for the parental species (Table 2). When looking to the differences between the models for the triplets involving *C. macromma* or *C. darwiniana*, we observed for the former a stronger migration rate coming from *C. arcania* (m13) and an admixture rate (f for HS scenario) close to 1, whereas for *C. darwiniana,* the results show higher values of gene flow coming from *C. gardetta* (m23) and an admixture rate of 0.4‐0.7 (Table 2).

### 3.2 Population structure

**Figure 4:**
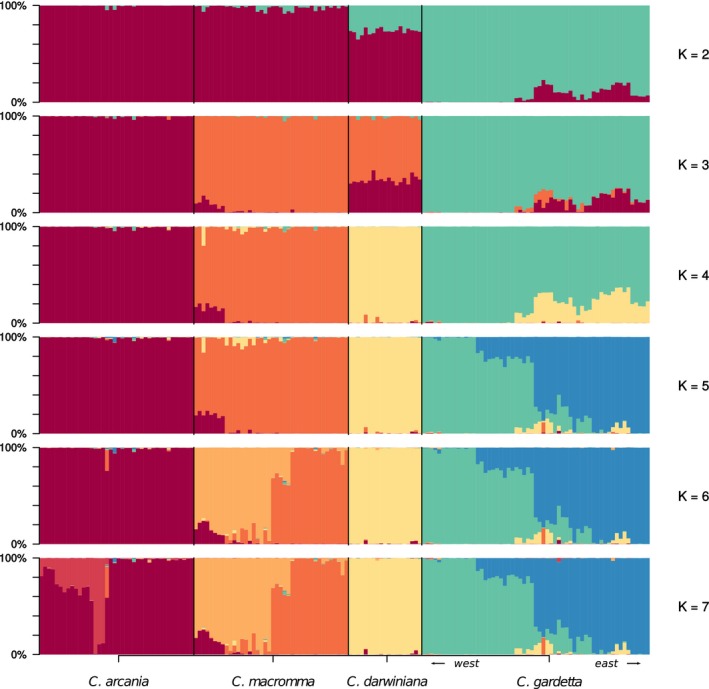
Genetic structure among and within the four studied species. The barplots indicate the probabilities of assignation of each individual to the K clusters ranging from 2 to 7 (obtained using STRUCTURE software). Only the allopatric populations have been used to produce this figure

**Figure 5:**
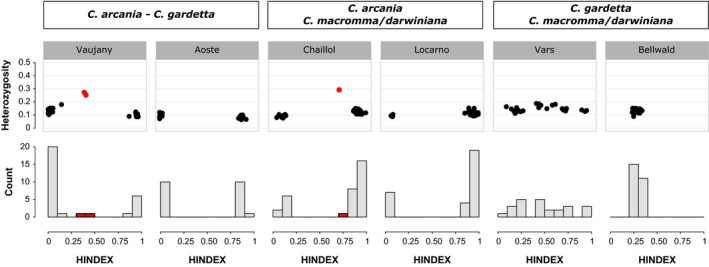
HINDEX distribution and interspecific heterozygosity in each studied contact zone. In red appear highlighted the individuals with admixed genetics genotypes (i.e., intermediate HINDEX) and a high heterozygosity

A hierarchic genetic structure is visible when increasing the number of potential genetic clusters across the allopatric populations of this group of butterflies (Figure 4). At *K* = 2, a split is observed between the alpine species (*C. gardetta*) and the rest of the sample. Interestingly, at *K* = 2 *C. darwiniana* shows high rates of admixture between the two genetic clusters*,* while *C. macromma* appears to have only a small contribution from the *C. gardetta* cluster. At *K* = 3, we observe the split of a hybrid species cluster (in orange) from *C. arcania*, followed by a split between *C. darwiniana* and *C. macromma* at *K* = 4 (orange and pale yellow in Figure 4). For K values higher than 4, we observe a genetic substructure consistent with within‐species geographic structuring. For instance, at *K* = 5, a split is observed between the *C. gardetta* individuals from the easternmost populations and the rest of this species. An interesting East‐West gradual admixture is visible between the two *C. gardetta* clusters, which is concordant with a pattern of isolation by distance (Appendix S2). For *K* = 6, *C. macromma* splits into two clusters, corresponding to the populations from the two sides of the Durance river valley in the southern French Alps. For higher values of *K* (>7), separate runs gave inconstant results, partitioning genetic composition of individuals in sub‐clusters lacking any consistency and with variable likelihood values for the independent runs (Figure 5; Appendix S3).

### 3.3 Strength of hybridization among species in contact zones

The analysis of the contact zones allows for the evaluation of the current degree of hybridization among the different taxa at a fine geographic scale. Six different locations have been investigated, corresponding to two replicates of the contact zone between parental species (*C. arcania*/*C. gardetta*) and one replicate of each of the other four possible parental/hybrid contact zones (Table 1, Figure 5). The results show different patterns depending on the species pair considered. Each time the lowland species is implicated (*C. arcania*), we observed a strongly bimodal distribution of individual assignation probabilities to genetic pools (HINDEX, Figure 5). In these four contact zones, *that is, C. arcania* with either *C. gardetta* (×2) or *C. macromma* or *C. darwiniana*, the distribution of individual HINDEX values is strongly bimodal, being close to 0 or 1, corresponding to one reference species or the other. However, at the contact zones Vaujany and Chaillol three individuals present a HINDEX between 0.5 and 0.7 (represented in red in Figure 5) and an interspecific heterozygosity around 0.3, much higher than the 0.1–0.2 observed for the rest of the samples (Figure 5), which suggests that they are recent hybrids between the reference species. Given their HINDEX values, two of them could be F1 or F2 hybrids between *C. arcania* and *C. gardetta* in Vaujany and the other could be a backcross between *C. arcania* and *C. macromma* in Chaillol.

The picture is completely different in contact zones between the alpine species (*C. gardetta*) and the two hybrid species (Figure 5). In the two contact zones investigated, only admixed individuals (HINDEX between 0.2 and 0.8) were observed and no “pure” *C. gardetta*,* C. macromma,* or *C. darwiniana* individuals were collected. Furthermore, the interspecific heterozygosity is low and rather similar for all the individuals in these two contact zones (~0.15), pointing out to two stable and long‐lasting hybrid zones, in the French Southern Alps for *C. gardetta*/*C. macromma* and in the Swiss Tessino for *C. gardetta*/*C. darwiniana*.

### 3.4 Genetic, morphological, and ecological differentiation among taxa

Patterns of genetic differentiation based on pairwise *F*_st_ values between species suggest that the two hybrid taxa are equidistant with regard to parental lineages (Table 3). Indexes of morphological distance, which are summarized in the Table 3, show a close correlation with the genetic differentiation among taxa. The highest morphological differentiation is observed between *C. arcania* and *C. gardetta,* whereas *C. macromma* is morphologically equidistant from its parental species and *C. darwiniana* is closer to *C. gardetta* than to *C. arcania*. In contrast, the Schoener's D close to 0 observed between *C. arcania* and the other taxa indicates almost no overlap between the climatic preferences of this lowland taxon and those of the three alpine species (Table 3). Indeed, the differences in altitudinal ranges of the four taxa place them into two divergent climatic envelopes: the three alpine species *vs*. the lowland species. Among the three alpine taxa, we observed values of Schoener's D around 0.5 for each pair of species, indicating highly overlapping climatic preferences (Table 3).

**Table 3:** Genetic differentiation (*F*_st_), morphological distance (Mahalanobis’ distances), and climatic niche similarity (Schoener's D metric) between each pair of species within the *Coenonympha* complex

	Genetic differentiation (*F*_st_)			Morphologic differentiation			Climatic niche similarity (D)		
	*C. macromma*	*C. darwiniana*	*C. gardetta*	*C. macromma*	*C. darwiniana*	*C. gardetta*	*C. macromma*	*C. darwiniana*	*C. gardetta*
*C. arcania*	0.29	0.32	0.44	27.1	51.6	78.9	0.007	0.006	0.006
*C. macromma*	–	0.17	0.32	–	15.4	33.8	–	0.51	0.46
*C. darwiniana*	–	–	0.29	–	–	16.7	–	–	0.54

## 4 DISCUSSION

The most significant result was our finding that hybridization has been pervasive throughout the speciation processes. Indeed, all the retained past demographic scenarios involved a significant degree of gene flow during the divergence and speciation of the four taxa, which led to the pattern of genetic differentiation observed within the complex today (Figure 3). However, by investigating the current genetic composition of individuals we found a striking asymmetry in gene flow among species. On one hand, an almost complete genetic isolation is now observable between the two hybrid lineages and one of their parental species, *C. arcania,* at any geographic scale considered, suggesting an advanced stage of the speciation process in that direction. On the other hand, the two hybrid lineages showed consequent genetic mixing with the other parental species *C. gardetta,* but only in contact zones, with no evidence of admixture at broad geographic scale. It suggests that complete isolation has not been achieved between these species, even if adaptive divergences certainly constrain their genetic backgrounds to remain distinct across their ranges of distribution.

### 4.1 Evolutionary scenarios of divergence and speciation

In a previous study, we suggested that *C. macromma* and *C. darwiniana* emerged from one common ancestral population originated through hybridization between *C. arcania* and *C. gardetta* (Capblancq et al., 2015). However, we did not consider then the possibility of variations in gene flow among diverging lineages. The present study tackles this issue by testing alternative demographic scenarios of divergence throughout the divergence processes, including possibilities of heterogeneous genetic exchange between species pairs (Figure 2).

#### 4.1.1 Divergence between parental species

The best and only evolutionary scenario retained for divergence between *C. arcania* and *C. gardetta* is an isolation with migration model of speciation involving recurrent migration after divergence (Figure 3a). This provides further support to a growing body of evidence suggesting that the IM model is the most probable scenario of speciation when the divergence is driven by ecological differentiation (Nosil, 2012; Rundle & Nosil, 2005), which appears to correspond with the biology of this pair of butterflies. The two species most likely diverged during the Pleistocene (1.5–4 million years ago according to Kodandaramaiah & Wahlberg, 2009 and Capblancq et al., 2015), probably concomitantly with the colonization and adaptation of *C. gardetta* to the harsher conditions of alpine environments (e.g., lower temperatures, stronger radiation, rapid annual turnover of plant communities). The other species, *C. arcania* is widely distributed throughout lowland Europe, and it rarely reaches elevations above 1500 m, after which it is replaced by *C. gardetta* (Lafranchis, 2000). Given that there is no obvious geographical separation in the distribution of the two species that could be considered as an isolating barrier, that they sometimes overlap in the 1,500–1,700 m altitudinal range, and that interspecific hybrids are occasionally found (Figure 4), we suggest that some form of disruptive selection associated with the ecological differences between low‐ and high‐elevation habitats must have contributed in the building‐up of reproductive isolation between these species. In addition, the IM model between *C. arcania* and *C. gardetta* is also consistent with the evidence that hybridization between these species led to the formation of the two hybrid taxa *C. macromma* and *C. darwiniana* as recently as during the last glacial maximum (Capblancq et al., 2015).

#### 4.1.2 Divergence of the hybrid species

In the case of the speciation process of *C. macromma* and *C. darwiniana*, the ∂a∂I approach struggles to decide between scenarios of speciation with secondary gene flow (SGF1 & SGF2) or of speciation through hybridization followed by consequent gene flow with both parental species (HS2P) (Figure 3b, Table 2 and Figure 2). These two families of speciation scenarios have been distinguished in the literature (Mavárez & Linares, 2008; Schumer et al., 2014) even if they both result in the production of a third new lineage or incipient species with mixed ancestry. In speciation with secondary gene flow, the new‐born species would have already begun to diverge from one of its parental species before receiving gene flow from the other parent, whereas in hybrid speciation the divergence of the new lineage would start concomitantly or after the hybridization between the two parental species (Mavárez & Linares, 2008; Schumer et al., 2014). Thus, depending on subtle variations in the timings of divergence and admixture, the speciation with secondary gene flow model becomes a hybrid speciation scenario and vice‐versa. In the case of this *Coenonympha* complex, we lack the required genetic resolution to confirm one of these scenarios with certitude. However, the best model returned by the analyses for both *C. macromma* and *C. darwiniana* suggests a scenario of speciation with secondary gene flow (SGF1, Figure 3b), involving first a split with the *C. arcania* population and then a history of divergence punctuated by consequent amounts of gene flow with both *C. arcania* and *C. gardetta* (Table 2). This scenario agrees with the *STRUCTURE* results showing an ancestral genetic cluster mostly grouping the hybrid species with *C. arcania* (*K* = 2 in Figure 4). It is also compatible with a common origin of *C. macromma* and *C. darwiniana*, from a single population*,* as proposed in the literature (Capblancq et al., 2015; Porter, Schneider, & Price, 1994; Wiemers, 1998).

It is worth noting that all the evolutionary reconstructions considered above suggest that hybridization has accompanied the differentiation of all the species in the complex. Furthermore, the best models of divergence always included heterogeneous gene flow along the genome, which means that some genomic regions do not cross the species boundaries, while others appear to be exchanged more easily. This suggests the action of selective pressures shaping gene flow between all species pairs. Only *C. macromma* and *C. darwiniana* appear to have ceased to exchange genes according to the best model retained (Figure 3a), which is consistent with the current allopatric distribution of the two lineages. Their ranges are more than 200 km apart, and it is highly likely that gene flow has now ended between them. The two hybrid lineages have nonetheless continued to experience different histories of gene flow with their parental species (Table 2), with *C. macromma* receiving more gene flow from *C. arcania*, and *C. darwiniana* more gene flow from *C. gardetta*. This is congruent with wing morphologies, which show a closer phenotypic proximity between *C. darwiniana* and *C. gardetta*, and between *C. macromma* and *C. arcania* (Table 3; Capblancq et al., 2015).

### 4.2 Inter‐ and intraspecific genetic structure within the species complex

The delimitation of species within this complex has already been assessed through genetic, morphologic and ecological analyses in a previous work (Capblancq et al., 2015). The four species are nonetheless confirmed by the clustering analyses performed with *STRUCTURE* in the present study: the first four differentiable genetic clusters corresponding to *C. arcania*,* C. gardetta*,* C. macromma,* and *C. darwiniana* (Figure 4). At *K* = 4, individuals are mainly associated with one of the four genetic groups and do not exhibit high rates of admixture, except for the easternmost *C. gardetta* individuals, which exhibit an admixed ancestry with *C. darwiniana*. However, this admixed ancestry almost completely disappears at *K* = 5, in which the *C. gardetta* cluster splits into two geographical groups reflecting an intraspecific genetic divergence congruent with isolation by distance from W to E of the Alps (Figure 4; Appendix S2). Thus, neither the past history of hybridization identified during the species divergences (Figure 3) nor the current hybridization events observed in the field (Figure 5) appear to induce a consequent mixing of the genetic backgrounds of the four species at large scale.

Some genetic structure is visible within taxa (Figure 4) and can probably be attributed to isolation by distance or geographic barriers (Figure 4; Appendix S2). Thus, as already mentioned, the *STRUCTURE* results at *K* = 5 highlights a gradual genetic transition between eastern and western populations of *C. gardetta* spread over several tens of kilometers along the Alps. On the other hand, at *K* = 6 a genetic divergence is observed between populations of *C. macromma* separated by a large river valley (Durance). With an elevation ranging from 750 to 950 m, this valley is probably low enough to limit the dispersal between populations of this alpine lineage. Topographic features (e.g., river valleys, mountain ranges) would thus interact with the intrinsic dispersion capacity of these species and be a determinant factor for the spatial scale at which genetic divergence can occur (Giordano, Ridenhour, & Storfer,^2007^; Kisel & Barraclough, 2010).

### 4.3 Reproductive isolation within the species complex

Although the species in this complex are genetically differentiated at large geographic scale, they are not entirely isolated reproductively and genetic exchanges can still occur, to a variable degree, in contact zones. The lowland species *C. arcania* is without doubt the most genetically isolated among the four. Its genetic exchanges with *C. gardetta* seem inexistent at broad spatial scale (Figure 4) and extremely limited in contact zones, in which we found only two recent hybrids (Figure 5). The small number of hybrids suggests that some strong pre‐ and/or postzygotic barriers to gene flow might be at play between this species pair. In the same way, strongly bimodal hybrid zones are also observed between *C. arcania* and the also alpine species *C. darwiniana* and *C. macromma* (e.g., in Locarno for *C. arcania*/*C. darwiniana* and in Chaillol for *C. arcania*/*C. macromma,* Figure 4). This suggests that isolation between *C. arcania* and the two hybrid lineages could be driven by processes similar to those involved in *C. arcania*/*C. gardetta* isolation.

A different pattern is found between the other parental species and the hybrid species, for whom we observe largely unimodal hybrid zones consisting almost entirely of admixed individuals between *C. gardetta* and *C. macromma* in Vars and between *C. gardetta* and *C. darwiniana* in Bellwald. This extensive mixing in contact zones suggests that prezygotic barriers to reproduction cannot be involved in the isolation between these species, in agreement with other studies suggesting that unimodal hybrid zones are associated mostly to extrinsic isolating factors related to climate, habitat, or biotic interactions (Gompert, Fordyce, Forister, Shapiro, & Nice, 2006; Jiggins & Mallet, 2000). Interestingly, the individuals captured outside the contact zone do not show any evidence of genetic introgression, even if located only a few kilometers away from the hybrid zones (Figures 1, 3).

### 4.4 Characterizing the progress of speciation

Among animals, most of the proposed hybrid species seem to show incomplete isolation with parental species (Brelsford, Milá, & Irwin, 2011; Hermansen et al., 2011; Kunte et al., 2011; Mavárez et al., 2006), but only few studies have really estimated the strength of this isolation (Mavárez et al., 2006 and Schwander, Suni, Cahan, & Keller, 2008). The investigations we have performed in this study point out to different degrees of isolation among parental and hybrid lineages within the species complex, suggesting that the speciation process has not completely been achieved among some of the species pairs. For instance, the two hybrid lineages seem to ceased gene flow with their lowland parental species *C. arcania*. Indeed, we found none or very few admixed individuals in contact zones among these species pairs (Figure 5) and only small traces of genetic admixture at larger scale (Figure 4). At the opposite, it seems that the two hybrid lineages remain genetically connected to their alpine parent *C. gardetta,* especially when their ranges of distribution abut and the species come into contact (Figure 5).

The necessity of isolation from parental populations during the first stage of a hybrid speciation would be one of the main selective pressures driving the parental trait reshuffling in hybrid populations (Mallet, 2007; Schumer et al., 2014). This should be particularly true for the recombination of traits already involved in parental reproductive isolation, and seems to have been the case within this *Coenonympha* species complex, in which the hybrid lineages would have kept the same isolating mechanisms against *C. arcania* than those that had already evolved in *C. gardetta*. In fact, the two hybrid lineages live in alpine climatic conditions similar to *C. gardetta,* which are very distinct from the ones preferred by *C. arcania* (Table 3). Therefore, the reproductive isolation of the hybrid lineages in regard to *C. arcania* is probably associated with their adaptations to the ecological conditions of life in high elevations, as for *C. gardetta*. During the hybridization swarm at the beginning of the hybrid speciation process, the ancestral population of lineages *C. darwiniana* and *C. macromma* would have retained the alpine ecological requirements from *C. gardetta*, and this might in turn have contributed to enhance the isolation with their lowland parental species *C. arcania*.

The case of the isolation between *C. gardetta* and the two hybrid lineages would require further work. At this time, it is unclear what drives differentiation among these taxa, but if ecological or geographic factors were the only or main drivers implicated (i.e., allopatry), future changes in local climate or habitats could greatly modify the distribution of species and have profound impacts on their current genetic integrity (Seehausen, Takimoto, Roy, & Jokela, 2008; Vonlanthen et al., 2012). A more precise investigation of contact zones between *C. gardetta* and the two hybrid lineages would be useful to assess the dynamic of genetic exchanges in such hybrid zones and their relation with environmental variations.

## AUTHOR CONTRIBUTIONS

Jesús Mavárez, Laurence Després, and Thibaut Capblancq designed the study. T.C. and D.R. performed ddRAD experiments. T.C. performed bioinformatics treatment of the resulted sequences, as well as the genetic analysis. T.C., J.M., and L.D. wrote the manuscript.

## CONFLICT OF INTEREST

None declared.

## Data Availability

Genetic data in the form of a table of genotypes for each individual and each retained site have been submitted to the Dryad Digital Repository under the accession number https://doi.org/10.5061/dryad.dq444fr. Morphologic data in the form of the coordinates of the 40 morphometric landmarks for each individual are available at the Dryad Digital Repository under the accession number https://doi.org/10.5061/dryad.j4389. A table with occurrence locations used for climatic niche comparisons is available at Dryad Digital Repository under the accession number https://doi.org/10.5061/dryad.j4389. Scripts used to performed the different analyses are freely available on Github at https://github.com/Capblancq/Speciation-Coenonympha-butterflies. Digitalized pictures of the butterfly wings are freely available upon request from T. Capblancq.
