# Supplementary figures and images for: Speciation with gene flow: Evidence from a complex of alpine butterflies (Coenonympha, Satyridae)
Source: Ecol Evol. 2019 May 3;9(11):6444–57. doi: 10.1002/ece3.5220 (PMC6580291; doi:10.1002/ece3.5220)

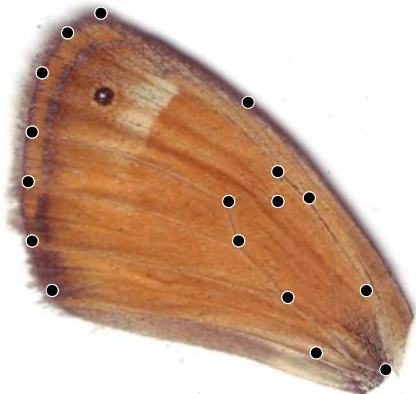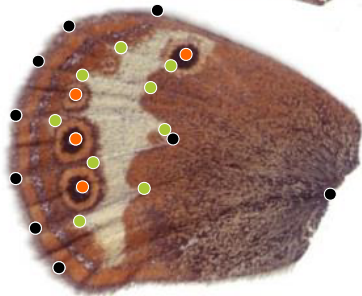

- White-band shape
- Eyespots alignment
- Venation

Supplement: Supplementary file 1 [file ECE3-9-6444-s001.pdf]

**C. gardetta**

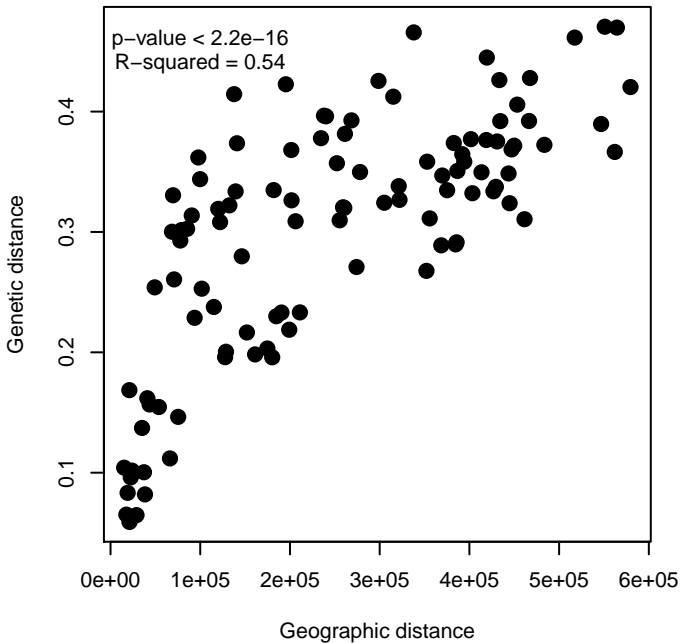

**C. darwiniana**

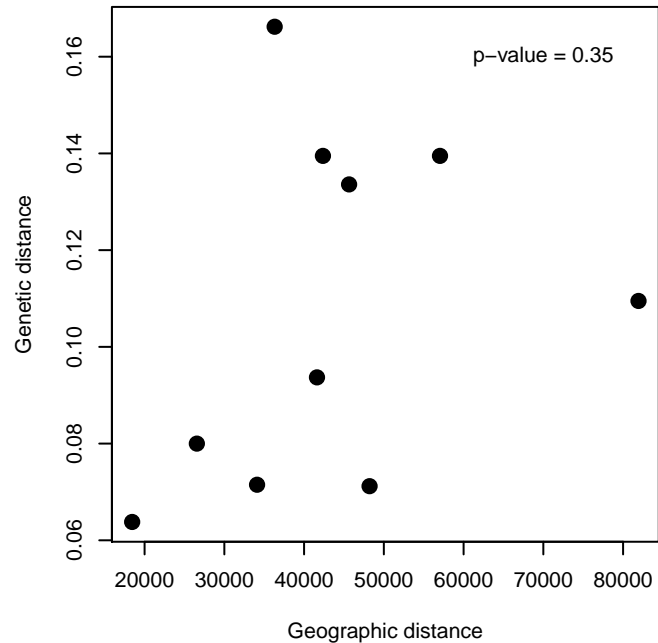

**C. macromma**

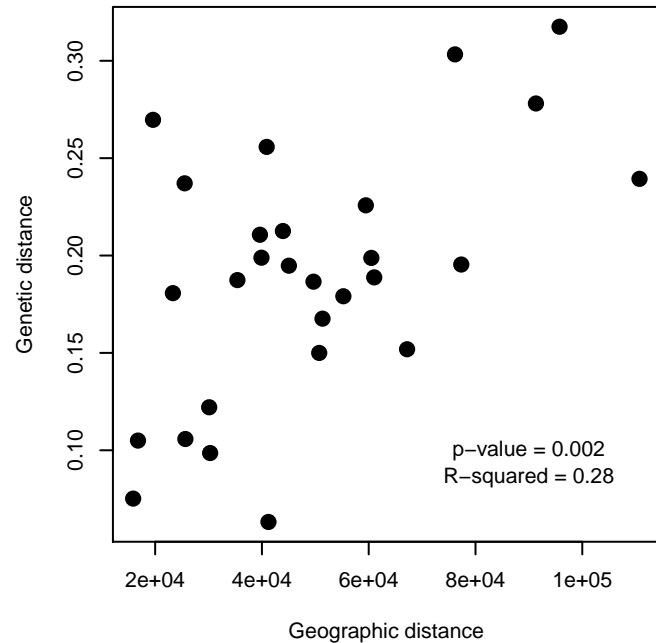

Supplement: Supplementary file 2 [file ECE3-9-6444-s002.pdf]

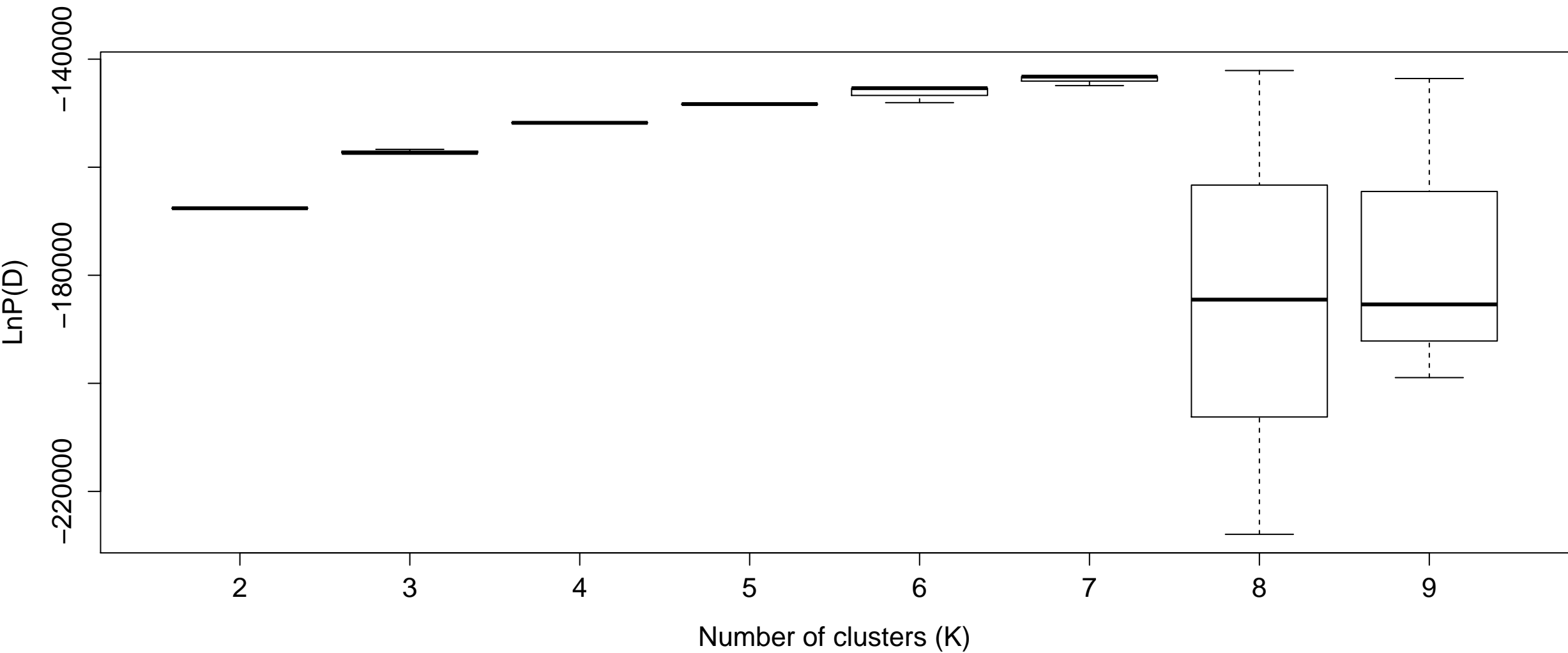

Supplement: Supplementary file 3 [file ECE3-9-6444-s003.pdf]
