# Supplementary material for: Speciation with gene flow: Evidence from a complex of alpine butterflies (Coenonympha, Satyridae)
Source: Ecol Evol. 2019 May 3;9(11):6444–57. doi: 10.1002/ece3.5220 (PMC6580291; doi:10.1002/ece3.5220)
Supplement: Supplementary file 4 [file ECE3-9-6444-s004.docx]

**Supporting information**

*Appendix S1:* The 40 landmarks used for the geometric morphometric analyses.

*Appendix S2:* Visualization of isolation by distance for allopatric populations of *C. gardetta, C. darwiniana,* or *C. macromma*. The significance tests were performed by regressing geographic distances with *Fst* between each pair of population (*lm* function of ade4 R-package).

*Appendix S3:* Likelihood of STRUCTURE runs with K ranging from 2 to 9.
